# LATINO HOSPICE PATIENTS’ INVOLVEMENT IN CARE DECISIONS AND PLANNING: FACTORS THAT SHAPE PARTICIPATION PREFERENCES

**DOI:** 10.1093/geroni/igz038.3376

**Published:** 2019-11-08

**Authors:** Susanny J Beltran

**Affiliations:** University of Central Florida, School of Social Work, Orlando, Florida, United States

## Abstract

Patient participation in care decisions is a primary tenet of patient-centered care and has been emphasized in policies and programs shaping clinical practice. Patient self-determination and involvement is associated with improved outcomes, including patient satisfaction and compliance. However, studies exploring Latino patients’ care involvement preferences find a cultural preference for limited autonomy and shared or family-based decision-making. This study describes terminally-ill older Latinos and their proxies’ preferences regarding involvement in decision-making and care planning following a referral to hospice care. Semi-structured interviews were conducted with 13 hospice-enrolled Latinos 65 or older, or their proxies. The sample is predominantly female (54% of all patients; 80% of proxies) and Mexican (77%). The average age of all patients was 82 years, and of proxies 54. Patients interviewed had an average of 7 years of education and proxies an average of 11 years. Patients were receiving care in a variety of settings, including inpatient hospice units, nursing homes, and community. Interviews were transcribed verbatim in the original language and coded thematically using an inductive approach. A second coder was used to code a Spanish and an English transcript (15% of the data), and a high level of interrater reliability was obtained (k = 0.85). Findings elucidate the paths through which culture, religion, and trust in provider shape preferences for limited autonomy and planning, confirming previous findings. However, this was not replaced by a preference for family-based decision-making. Findings highlight the importance of Latinos having access to culturally-competent primary care providers and fostering lasting relationships.

